# The impact of the EU general data protection regulation on scientific research

**DOI:** 10.3332/ecancer.2017.709

**Published:** 2017-01-03

**Authors:** Gauthier Chassang

**Affiliations:** INSERM UMR 1027, Toulouse F-31000, France; INSERM US 13—Infrastructure BIOBANQUES, Paris F-75651, France; Université Paul Sabatier, Toulouse F-31000, France

**Keywords:** privacy, computer security, humans, European Union (EU), translational medical research, biomedical research

## Abstract

The use of personal data is critical to ensure quality and reliability in scientific research. The new Regulation [European Union (EU)] 2016/679 of 27 April 2016 on the protection of natural persons with regard to the processing of personal data and on the free movement of such data [general data protection regulation (GDPR)], repealing Directive 95/46/EC, strengthens and harmonises the rules for protecting individuals’ privacy rights and freedoms within and, under certain conditions, outside the EU territory. This new and historic legal milestone both prolongs and updates the EU acquis of the previous Data Protection Directive 95/46/EC. The GDPR fixes both general rules applying to any kind of personal data processing and specific rules applying to the processing of special categories of personal data such as health data taking place in the context of scientific research, this including clinical and translational research areas. This article aims to provide an overview of the new rules to consider where scientific projects include the processing of personal health data, genetic data or biometric data and other kinds of sensitive information whose use is strictly regulated by the GDPR in order to give the main key facts to researchers to adapt their practices and ensure compliance to the EU law to be enforced in May 2018.

## Introduction

After a long and intense reform, the European Union (EU) adopted the new Regulation (EU) 2016/679 of the European Parliament and of the Council of 27 April 2016 [1] on the protection of natural persons with regard to the processing^1^ of personal data^2^ and on the free movement of such data [general data protection regulation (GDPR)], repealing the previous Data Protection Directive 95/46/EC of 1995 [2]. With the GDPR, the EU reaffirms its attachment to the protection of fundamental rights and freedoms of individuals, notably those related to the protection of individuals’ privacy including the specific fundamental right to personal data protection enshrined within the Charter of the Fundamental Rights of the EU^3^ [3] and within the primary EU law Treaty on the Functioning of the EU^4^ [4], as well as its willingness to accelerate the achievement of the internal market for which the free flow of personal data is essential, for commercial and non-commercial relationships. The GDPR aims to harmonise the rules for all the Member States in order to reduce the legal fragmentation, complexities and uncertainties that existed between Member States under the Data Protection Directive, and to reinforce the data subjects’^5^ rights in a digitalised and evolutive environment in order for them to regain control over their personal data. The ultimate goal of the GDPR is to create legal certainty and sustainability of the data protection measures in a technological neutral^6^ approach. Without fundamentally changing the approach to the field compared to what existed previously with the Directive of 1995, the GDPR performs several updates and introduces some new individual rights and procedures of importance which impact scientific research activities. Indeed, the GDPR still applies roughly to the data controllers^7^ and processors^8^ acting in the public and private sectors for profitable and not-profitable purposes. It still differentiates between two kinds of personal data by strictly regulating the processing of special categories of data (the so-called ‘sensitive personal data’ such as health data, genetic data and biometric data) because of their potential risks regarding the rights and freedoms of the data subject. It will still also consider scientific research^9^ activities as a specific context of personal data processing where the equilibrium between individual freedom and the freedom of research triggers particular challenges and ethical issues^10^, thus necessitating appropriate rules^11^ allowing both personal data processing and sharing in the pursuit of the public interest. The GDPR adopts a new general risk-based approach intended to facilitate the case-by-case identification of data protection issues and the related necessary data protection measures to respect. Because personal data processing and the use of sensitive personal data^12^ such as genome-based information are crucial for the advances of health research activities such as clinical research and translational research, for practicing whole genome sequencing, for research biobanking or the creation of research databases, this article describes the main news^13^ or specifications of importance concerning scientific research activities that have to be considered in the coming years. We address, in particular, the following: (1) the new general principles to apply in research settings, (2) the new important legal terminology for research, (3) the new procedures to respect, (4) the specific provisions regarding data subjects’ rights in the context of research and (5) the new opportunities to further regulate data protection in the field.

## The strengthening of the general principles applying to any personal data processing for scientific research purposes

The GDPR maintains the approach of the previous Directive by fixing general principles to be observed in any context of personal data processing, including in research and for archiving purposes in the public interest, and regardless of the kind of personal data, including to the processing of data qualified as sensitive personal data. Nevertheless, the GDPR adds three new general principles of importance.

The main general principles remain the same than under the previous Directive. Indeed, according to Article 6 of the GDPR, personal data shall be processed lawfully, fairly and in a transparent^14^ manner in relation to the data subject^15^; collected for specified, explicit and legitimate purposes and not further processed in a manner that is incompatible with those purposes^16^; adequate, relevant and limited to what is necessary in relation to the purposes for which they are processed^17^; accurate and, where necessary, kept up to date; every reasonable step must be taken to ensure that personal data that are inaccurate, having regard to the purposes for which they are processed, are erased or rectified without delay^18^ ; kept in a form which permits identification of data subjects for no longer than is necessary for the purposes for which the personal data are processed^19^.

The GDPR completes the principles cited above by fixing two relatively new additional principles of general application in its Article 6 which existed under the previous Directive but which have now acquired a new dimension. The first principle is about respect of the data integrity and of their confidentiality. This principle imposes that the data be processed in a manner that ensures appropriate security of personal data, including protection against unauthorised or unlawful processing and against accidental loss, destruction or damage^20^, using appropriate technical or organisational measures. This principle will find application not only through the enforcement of health professional rules and research ethics guidelines, such as those ensuring scientific and research integrity^21^ [5], but also through technical measures, such as the use of coding techniques (e.g. pseudonymisation, cryptography or anonymisation technics), the use of protected servers against external threats, closed-controlled system of data processing etc. This principle is particularly important in the research context where a potentially large amount of sensitive data are at stake and where the quality of the data is essential to ensure research results to be reliable, verifiable and useful. The second principle which was also existing under the previous Directive but that has been clarified and associated with detailed implementation procedures (see below) is the accountability principle. According to this principle, the controller shall be responsible for, and be able to demonstrate compliance with the general principles of data processing exposed above. This will necessitate, in particular, that the controller, or where applicable its representatives in the EU, and the processors organise and maintain clear and secured records of any data processing activities performed under their responsibility in order to be able to demonstrate compliance with the GDPR. In research, such records can constitute archives to be retained for a certain period of time according to applicable law. The GDPR explicitly details the minimal information to be preserved within such records in its Article 30. Each controller and processor should be obliged to cooperate with the supervisory authority and make those records available, on request, so that it might serve for monitoring those processing operations.

Another principle of general application has appeared distinctly from the ones established under Article 6, namely the principles of ‘data protection by design and by default’ (DPbD/Dflt) fixed in Article 25. This new principle traduces the integrated approach adopted by the EU in order to create a sustainable data protection system through the early use of adapted privacy enhancing technologies^22^ [6, 7] in the design of the processing operations and throughout the life cycle of the data. Here, the EU definitely approximates the law and the technology, two essential elements of the data protection system that shall develop together to allow legal compliance in a modern world. According to this techno-legal approach, the GDPR states that ‘considering the state of the art, the cost of implementation and the nature, scope, context and purposes of processing as well as the risks of varying likelihood and severity for rights and freedoms of natural persons posed by the processing, the controller shall, both at the time of the determination of the means for processing and at the time of the processing itself, implement appropriate technical and organisational measures, such as pseudonymisation, which are designed to implement data-protection principles, such as data minimisation, in an effective manner and to integrate the necessary safeguards into the processing in order to meet the requirements of this Regulation and protect the rights of data subjects.’ This data protection by design approach is something well known in scientific research settings, notably in the context of funding application where the applicant have to demonstrate the robustness of the data protection system to be implemented in the course of the research in order to be granted. The technical aspects are completed by organisational measures which allows data protection to be respected (e.g. data management plan, privacy policies and instructions etc). However, this by-design feature is completed by the default one. With this latter, ‘the controller shall implement appropriate technical and organisational measures for ensuring that, by default, only personal data which are necessary for each specific purpose of the processing are processed. That obligation applies to the amount of personal data collected, the extent of their processing, the period of their storage and their accessibility. In particular, such measures shall ensure that, by default, personal data are not made accessible without the individual’s intervention to an indefinite number of natural persons.’ The by-default feature has the specificity that the system alone should ensure sufficient protection without any human action. However, it should not be entirely autonomous, as it would create a risk in terms of data control and thus a loss of the protective feature of the by-default criterion. An approved certification mechanism pursuant to Article 42 of the GDPR may be used as an element to demonstrate compliance with the requirements regarding DPbD/Dflt.

In addition to these principles, Article 9 of the GDPR fixes general rules regarding respect for processing sensitive personal data, such as data concerning health or genetic data, and keeps the previous mechanisms based on a general prohibition^23^ of processing with some important exceptions^24^, in particular, for the healthcare practice and the management of health systems^25^, public health^26^ and research^27^ sectors where the processing is authorised under specific conditions. Focusing on research, according to Article 9 al.2 (j), the processing of personal sensitive data for archiving purposes in the public interest, scientific or historical research purposes or statistical purposes shall be necessary, for the benefit of natural persons and society as a whole, and based on Union or Member State law ‘which shall be proportionate to the aim pursued, respect the essence of the right to data protection and provide for suitable and specific measures to safeguard the fundamental rights and the interests of the data subject.’ Here, we can note the new importance of pursuing a purpose of public interest for justifying the processing of sensitive personal data, as it is clearly explicated in Recitals 53 and 54. In addition, the data controller shall respect the new Article 89(1) of the GDPR requiring both sufficient and adequate technical and organisational measures ensuring data protection and, in particular, in this context, the respect of data minimisation.

Beside these fundamental provisions, the GDPR also introduce new important definitions that will base further interpretation of the GDPR.

## The new legal definitions of importance for using personal data in scientific research

Understanding the legal terminology is paramount for ensuring its proper dissemination and application by stakeholders. In the field of research, lawyers met difficulties in understanding and circumventing notions which are very scientifically based and depend on the evolution of technologies and contexts. With the GDPR, we can salute the work that has been done by the EU legislator to design several definitions of direct utility in the context of scientific research and that represent the new common benchmark for the Member States. In particular, the GDPR introduces some new definitions of certain special categories of personal data whose processing is forbidden, by principle, but exceptionally admitted for research or archiving purposes in the public interest in the respect of Articles 9 and 89 of the GDPR.

- ‘Data concerning health^28^’ means personal data related to the physical or mental health of a natural person, including the provision of health care services, which reveal information about his or her health status. A Recital^29^ provides further details about what shall be considered as personal health data under the GDPR, and we can see that this notion is inclusively defined as ‘all data pertaining to the health status of a data subject which reveal information relating to the past, current or future physical or mental health status of the data subject. This includes information about the natural person collected in the course of the registration for, or the provision of, health care services as referred to in Directive 2011/24/EU of the European Parliament and of the Council [8] to that natural person; a number, symbol or particular assigned to a natural person to uniquely identify the natural person for health purposes; information derived from the testing or examination of a body part or bodily substance, including from genetic data and biological samples; and any information on, for example, a disease, disability, disease risk, medical history, clinical treatment or the physiological or biomedical state of the data subject independent of its source, for example, from a physician or other health professional, a hospital, a medical device or an in vitro diagnostic test.’
- ‘Genetic data^30^’ means personal data relating to the inherited or acquired genetic characteristics of a natural person which give unique information about the physiology or the health of that natural person and which result, in particular, from an analysis of a biological sample from the natural person in question. In addition to this, a Recital^31^ specifies that genetic data can consist of ‘result from the analysis of a biological sample from the natural person in question, in particular chromosomal, deoxyribonucleic acid (DNA) or ribonucleic acid (RNA) analysis, or from the analysis of another element enabling equivalent information to be obtained.’ The very last part of this Recital can trigger issues regarding the boundaries of the notion of genetic data which should be restrictive instead of extensive. With this opening, it is hard to understand what kind of data could potentially be qualified as genetic data. Could this be genealogical information gathered through questionnaires? Could this aims epigenetic data? To a certain extent, this creates confusions with regard to the notion of biometric data (see below). Whatever, this definition seems to be a very useful and workable basis.
- ‘Biometric data^32^’ means personal data resulting from specific technical processing relating to the physical, physiological or behavioural characteristics of a natural person, which allow or confirm the unique identification of that natural person, such as facial images or dactyloscopic data.

Regarding the condition of the data, the GDPR also adopts new definitions, those of pseudonymisation and encryption, and confirms the previous notion of anonymous data.

- ‘Pseudonymisation^33^’ means the processing of personal data in such a manner that the personal data can no longer be attributed to a specific data subject without the use of additional information, provided that such additional information is kept separately and is subject to technical and organisational measures to ensure that the personal data are not attributed to an identified or identifiable natural person. The result of pseudonymisation is pseudonymised data which remain personal data but being protected through coding or encryption. Throughout the GDPR, the use of pseudonymisation is promoted and shall be implemented, as far and as soon as possible, in personal data processing for scientific research purposes, as a standard data protection practice.
- ‘Anonymous data^34^’ are defined as information which does not relate to an identified or identifiable natural person or to personal data rendered anonymous in such a manner that the data subject is not or no longer identifiable. This Regulation does not, therefore, concern the processing of such anonymous information, including for statistical or research purposes.

In addition, among the issues surrounding the rights of the data subjects, the GDPR provides a new definition of the consent term.

- ‘Consent^35^’ of the data subject means any freely given, specific, informed and unambiguous indication of the data subject’s wishes by which he or she, by a statement or by a clear affirmative action, signifies agreement to the processing of personal data relating to him or her. Here, we can note that the notion has been specified regarding the unambiguous feature of the consent which does not rise doubt about the scope of the activities agreed by the data subjects and regarding the form of consent that shall be a statement or by a clear affirmative action. The new rules regarding consent of the data subject, including in the research field, are further exposed below.

Some could regret that the GDPR does not define notions such as ‘big data’ or ‘cloud computing’ which are very often used in debates around data protection without harmonised legal definition.

Aside from these terminological advances, the GDPR innovates in the way data protection will be ensured in practice by setting up some new procedures to adhere to.

## The new procedures of importance for using personal data in scientific research

The GDPR establishes a new system using a risk-based approach. This new approach is implemented through an integrated system of data protection, both close to the data controller(s) and processors, and able to adapt to the diverse contexts of processing. Thus, this system relies on a couple of new procedures which are also applied in scientific research settings.

### The designation of a data protection officer

The procedure of designating a data protection officer (DPO) by the data controller is a very structuring point. In most of the case for research organisations, biobanks, and health database infrastructures as well as in the context of most of research projects, the DPO designation will be mandatory. Indeed, according to Article 37, designating a DPO is mandatory where the processing is carried out by a public authority or body, except for courts acting in their judicial capacities, or where the core activities of the controller or the processor consist of either processing operations which, by virtue of their nature, their scope and/or their purposes, require regular and systematic monitoring of data subjects on a large scale; or where the processing concerns sensitive personal data and is on a large scale. An important criterion here is the scale of the processing. However, the GDPR does not define when a processing shall be considered as a ‘processing on a large scale’ what is quite problematic for the implementation of this article. Recital 91 of the GDPR dealing with the data protection impact assessment (DPIA) refers to large-scale processing operations ‘which aim to process a considerable amount of personal data at regional, national or supranational level and which could affect a large number of data subjects.’ Looking back to a previous official version of the GDPR amended by the EU Parliament [9] in 2014, this notion appeared more clearly in the context of an article about risk assessment (cf to the DPIA section below) as targeting ‘processing of personal data relating to more than 5000 data subjects during any consecutive 12-month period^36^.’ While the current version of the GDPR seems less explicit on this point, it also introduces a troublesome provision in its Recital 91 mentioning, also regarding the DPIA, that ‘the processing of personal data should not be considered to be on a large scale if the processing concerns personal data from patients or clients by an individual physician, other health care professional or lawyer.’ It is hard to understand such a focus on specific occupations as it seems relevant to assess the size of the processing whatever the context for ensuring appropriate data protection. Furthermore, the GDPR does not consider activities such as full genome sequencing or the use of other high throughput technologies in health and research sectors as being on a large scale despite the fact that such a process creates big data at the individual level and particular risks regarding privacy protection^37^ [10].

Where a DPO has to be mandatorily designated, its designation shall be based on professional qualities and expert knowledge of data protection law and practices in the field. This will necessitate adequate educational programs with teaching in law and ethics. The DPO shall show sufficient skills to perform its tasks fixed in Article 39. The DPO role will be central in the organisations as it has to inform and advise the controller or the processor on their obligations, to monitor compliance with the GDPR, to cooperate with the national data protection authority (NDPA) as a privileged contact point (or ‘supervisory authority’). In that sense, the data controller and the processor will have to properly and timely consult the DPO in the decision-making process regarding data protection issues. In addition, the DPO may be the contact for data subjects’ willing to exercise their rights according to the GDPR. According to Article 37(6), ‘the DPO may be a staff member of the controller or the processor, or fulfil the tasks on the basis of a service contract,’ but whatever the case, the DPO is bound to secrecy and confidentiality as imposed by the EU or national laws. As stated in Article 38, the DPO shall have necessary means to perform its tasks independently, without receiving any instructions from the controller or processor for performing its tasks. In addition, a DPO shall not be dismissed or penalised by the controller or the processor for performing his tasks. The DPO shall directly report to the highest management level of the controller or the processor. Therefore, this new procedure should not be considered as an additional burden but as an initial investment to bring data protection governance closer to the organisations’ needs, for making data protection a daily practice. An EU DPO network should emerge in support of a harmonised approach of practices.

### The practice of the data protection impact assessment

With the GDPR, research sponsors and investigators, as data controllers, will certainly have to practice a DPIA according to Article 35 of the GDPR. The DPIA is an entirely new self-assessment exercise which somewhat prolongs the requirements of most of the funding agencies requiring, as an integrated part of the ethics assessment of a research proposal, to describe how personal data will used and responsibly managed in the research (e.g. in H2020 or ERC programs). The DPIA concretises the risk-based approach of the GDPR. The aim of the DPIA is to assess the likelihood and severity of the risk regarding data subjects’ rights and freedoms before undertaking the processing. The DPIA serves not only to know the state of the art of data protection means in a certain context, to plan and manage the necessary enhancements to ensure compliance of the system, but also to determine if a prior consultation^38^ of the supervisory authority is necessary. Indeed, the GDPR abolishes the obligation to systematically declare any kind of personal data processing in favour of the sole declaration of the processing that is likely to result in a high risk to the rights and freedoms of data subjects. This concerns, in particular, situations where new technologies are used (we can think about full genome sequencing), where special categories of data is processed on a large scale^39^ (presumably in biobanking and research cohorts) or in the context of a systematic and extensive evaluation of personal aspects relating to natural persons which is based on automated processing, including profiling activities, on which decisions are based that produce legal effects concerning the natural person or similarly significantly affect the natural person (such as for some e-health technologies). The NDPAs shall establish and publish lists of processing for which a mandatory DPIA is required, after assessment and approval from the new European Data Protection Board (EDPB) in application of the new consistency mechanism^40^. The GDPR details *a minima* the information that the assessment’s results must contain. Indeed, according to Article 35(7) of the GDPR, ‘the assessment shall contain at least: a systematic description of the envisaged processing operations and the purposes of the processing, including, where applicable, the legitimate interest pursued by the controller; an assessment of the necessity and proportionality of the processing operations in relation to the purposes; an assessment of the risks to the rights and freedoms of data subjects […]; and the measures envisaged to address the risks, including safeguards, security measures and mechanisms to ensure the protection of personal data and to demonstrate compliance with this Regulation considering the rights and legitimate interests of data subjects and other persons concerned.’ The nominated DPO has to be involved in the DPIA process. The controller shall consult the supervisory authority prior to processing where the results from the DPIA indicate that the processing would result in a high risk in the absence of measures taken by the controller to mitigate the risk^41^. The controller shall review the DPIA, in particular, where a significant change occurred in the processing that change the nature or scope of the risk generated by the processing^42^. DPIA reports will need to be recorded and made available to the authorities in accordance with the accountability principle. Some well-known existing methodologies [11] elaborated at national level before the adoption of the GDPR provide a good idea of the practice of the DPIA and tools. These methodologies can still be used today as they keep on complying with the GDPR. The new EDPB also has a competency to propose European guidelines on this exercise^43^.

### Rules regarding the reuse of personal data for research purposes

It is a daily practice in scientific research that personal data for a purpose that is different from the initial collection one (also called ‘secondary use’ or ‘further processing’) of personal data processed otherwise. Allowing this kind of processing is crucial as the access to personal data that can be reused for different objectives constitutes an essential activity for scientific and translational research.

As a general principle stated under Article 5 of the GDPR (see above), the processing of personal data for purposes other than those for which the personal data were initially collected should only be allowed where the new purpose of the processing is compatible with the purposes for which the personal data were initially collected. Here, the GDPR preserves the previous approach of the Directive of 1995 planning that ‘further processing for archiving purposes in the public interest, scientific or historical research purposes or statistical purposes should be considered to be compatible lawful processing operations,’ where Article 89(1) is respected. This presumption of compatibility with the initial purposes of the processing advanced at the time of the first collection is notably related to the specific exemption to the principle of storage minimisation, where the further processing (e.g. storage) is for research or archiving purposes in the public interest^44^. This is a good news for health registries, cohorts and research biobanking maintaining personal sensitive data available for future scientific or statistical reuses. However, this presumed compatibility is not fully automatic and must answer to several requirements such as the respect of data minimisation principle. Indeed, according to Article 89(1) and Recital 156, this further processing ‘is to be carried out when the controller has assessed the feasibility to fulfil those purposes by processing data which do not permit or no longer permit the identification of data subjects (e.g. pseudonymisation of the data), and provided that appropriate safeguards exist [e.g. secured and separated storage of the identifiers (codes) and respect of the relevant ethical standards in the field].’

In other cases of reuses, where the processing for another purpose is not based on the data subject’s unambiguous consent or on an EU or Member State law, the controller shall perform a purpose compatibility test^45^. This test is a new and very useful tool providing criteria that the controller shall use in order to ascertain whether processing for another purpose is compatible with the purpose for which the personal data are initially collected. According to Article 6(4), the controller willing to reuse the data will have to consider, *inter alia*, ‘any link between the purposes for which the personal data have been collected and the purposes of the intended further processing; the context in which the personal data have been collected, in particular regarding the relationship between data subjects and the controller; the nature of the personal data, in particular whether special categories of personal data are processed’ […], or whether personal data related to criminal convictions and offences are processed […]; the possible consequences of the intended further processing for data subjects; the existence of appropriate safeguards, which may include encryption or pseudonymisation. Where the results of the test shows that none of these elements has significantly changed in a way that would make the further processing unfair or otherwise illicit, the compatibility test is satisfied and no legal basis separated from that which allowed the initial collection of the personal data is required. If not, the further processing will have to rely on a separate legal basis (e.g. re-consent of the individual).

### Notification and communication of personal data breach

The main rationale of any regulation on personal data aims to avoid the realisation of privacy risks, namely the occurrence of the so-called ‘personal data breach’ defined as ‘a breach of security leading to the accidental or unlawful destruction, loss, alteration, unauthorised disclosure of, or access to, personal data transmitted, stored or otherwise processed^46^.’ Because ‘a personal data breach may, if not addressed in an appropriate and timely manner, result in physical, material or non-material damage to natural persons, such as loss of control over their personal data or limitation of their rights, discrimination, identity theft or fraud, unauthorised reversal of pseudonymisation, damage to reputation, loss of confidentiality of personal data protected by professional secrecy or any other significant economic or social disadvantage^47^,’ the GDPR developed new and stricter notifications rules for the controller and the processor, focusing on the need to be rapid and efficient, in the respect of transparency. Indeed, as soon as a breach has been noticed, in parallel of the relevant corrective actions to immediately undertake, the processor shall notify the controller without undue delay. Then, two kinds of actions must be implemented: (1) a notification^48^ of the competent supervisory authority, including through the DPO and (2) in limited cases, a communication^49^ with the concerned data subjects.

First, according to Article 33, whatever the nature, scope and context of the breach, the controller shall, as soon as he/she becomes aware that a personal data breach has occurred, notify the personal data breach to the supervisory authority without undue delay and, where feasible, not later than 72 h after having become aware of it, unless the controller is able to demonstrate, in accordance with the accountability principle, that the personal data breach is unlikely to result in a risk to the rights and freedoms of natural persons. Where such notification cannot be achieved within 72 h, the reasons for the delay should accompany the notification and information may be provided in phases without undue further delay. The minimal content of the notification to address to the NDPA is specified in Article 33(3) and includes notably a description of the facts, of the nature of the breach, of the categories and approximate number of both the data subjects concerned and the records affected by the breach, an analysis of the likely consequences of the breach and the measures taken or proposed by the controller to address the personal data breach and, where appropriate, to mitigate possible adverse effects. Where the information required cannot be entirely provided at the same time, the GDPR allows providing it in several phases.

Second, without prejudice to the previous obligation, in application of Article 34, only ‘when the personal data breach is likely to result in a high risk to the rights and freedoms of natural persons, the controller shall communicate the personal data breach to the data subject without undue delay.’ This obligation has a number of exceptions^50^ notably where the controller ‘has implemented appropriate technical and organisational protection measures’ to stop or palliate the breach and, in particular, where those actions rendered unintelligible the personal data concerned for those who does not have authorised access (e.g. through encryption) or where this communication would involve disproportionate efforts. In the latter case, individual communication shall be replaced by general public information about the breach or a similarly effective communication mean that would easily allow the data subjects to be aware of the facts and consequences of the breach. Where the communication with the data subjects is to be done, it must be transparent and presents information in clear and plain language. The point of this communication with the data subjects is to provide him with both informative and useful information such as advices on the way to act. A supervisory authority, mandatorily notified in the respect of Article 33, can, having considered the likelihood of the personal data breach resulting in a high risk, require to implement such communication with the data subjects or decide that any of the exemption conditions^51^ are met.

In a dedicated article, the GDPR also now clearly deals with the implementation of individuals’ data protection rights where the processing is performed for research purposes.

## The new provisions related to research participants’ rights

The GDPR fixes some new provisions specifying the application of the data subjects’ rights in the specific context of archiving in the public interest and scientific historical or statistical research. These are essentially concentrated in Article 89 of the GDPR but not only.

### About the individual consent to the processing of personal data for research purposes

Consent has always been a central ethical element for participating in research projects involving human beings. New research practices triggered debates in Europe about the risk that the GDPR require systematic consent before each and every data processing and around the necessity to allow the practice of broad consent intended to maximise the use of personal data, including sensitive data for several different and unknown research purposes. We will successively address these questions.

Regarding the fear of seeing written consent becoming a systematic obligation for processing personal data for research uses, the GDPR keeps consent as only one of the means justifying the lawfulness of the processing^52^. Other legal grounds exposed within Article 6 (for personal data) and Article 9 (for sensitive personal data) of the GDPR can be invoked to legitimate such a processing. Here, it is particularly important to remind that the GDPR has to articulate with other relevant EU laws in the field, such as the clinical trail regulation (CTR) [12] of 2014 and other *lex specialis*, including at national level, which require consent. The GDPR also now explicitly recognises that ‘the processing of special categories of personal data may be necessary for reasons of public interest in the areas of public health without consent of the data subject. Such processing should be subject to suitable and specific measures so as to protect the rights and freedoms of natural persons. In that context, ‘public health’ should be interpreted as defined in Regulation (EC) No 1338/2008 of the European Parliament and of the Council [13], namely all elements related to health, namely health status, including morbidity and disability, the determinants having an effect on that health status, health care needs, resources allocated to health care, the provision of, and universal access to, health care as well as health care expenditure and financing, and the causes of mortality. Such processing of data concerning health for reasons of public interest should not result in personal data being processed for other purposes by third parties such as employers or insurance and banking companies^53^.’

Another important concern of the scientific community with the GDPR was to see the practice of broad consent invalidated^54^. Taking clinical trials as an example, the consent process is well described through the CTR and the protocol has to answer to specific criteria to be reviewed by research ethics committees. The CTR allows the practice of broad consent^55^ as far as the national laws does not require otherwise in a particular research context. For other kinds of research, the definitive version of the GDPR explicitly recognises that ‘it is often not possible to fully identify the purpose of personal data processing for scientific research purposes at the time of data collection. Therefore, data subjects should be allowed to give their consent to certain areas of scientific research when in keeping with recognised ethical standards for scientific research. Data subjects should have the opportunity to give their consent only to certain areas of research or parts of research projects to the extent allowed by the intended purpose^56^.’ To this broad approach of the consent to personal data processing research, the GDPR adds, regarding the lawfulness of the processing, that the data subjects’ consent can be done for ‘one or more specified purposes,’ thus allowing broad consent in the respect of applicable national law, provided that the individual received sufficiently clear information and that the given consent represents the unambiguous indication of the data subject’s wishes.

### Specific exemptions regarding other data subjects’ rights in research

Research participants, as data subjects, have several rights allowing them to maintain a certain degree of control over their personal data processed in the course of the research. While there was hope for achieving a new level of harmonisation on this topic, the GDPR is quite deceiving as, even if it fixes new important rights of general application such as the right to be forgotten or the right to data portability, they could not apply in the field of research, if the EU or member States laws provides, under certain conditions, legitimate exceptions, as it is stated under Article 89. This situation is mainly due to an absence of conferred competency to the EU to harmonise legislations in the field of health and scientific research, the EU having only a support competency in these fields remaining principally regulated by national laws. This results in the incapacity for the EU to adopt fully harmonised rules through EU law without the formal agreement of EU Member States, this explaining the limited content of Article 89 that fixes rules depending on the state of the art of national or EU laws. Thus, Member States should provide for appropriate safeguards for the processing of personal data for archiving purposes in the public interest, scientific or historical research purposes or statistical purposes. Member States should be authorised to provide, under specific conditions and subject to appropriate safeguards for data subjects, specifications and derogations with regard to the rights to rectification, to erasure, to be forgotten, to restriction of processing, to data portability and to object. The conditions and safeguards in question should ensure that technical and organisational measures are in place in order to ensure, in particular, the principle of data minimisation, and may entail specific procedures for data subjects to exercise those rights if this is appropriate in the light of the purposes sought by the specific processing, in pursuance of the proportionality and necessity principles. The respect of relevant and recognised ethical standards as well as the requirements for obtaining ethical approvals is part of these safeguards.

## The new opportunities for standardising data protection practices in research

The GDPR enhances the means available to develop accountability based on self-regulations, in particular, through the new incentives to elaborate sectorial Code of Conducts^57^, binding corporate rules^58^ (for private multinational undertakings) and to create data protection seals^59^. To be effective, these tools have to be validated according to special procedures laid down by the GDPR. These tools are interesting for research communities looking for more standardisation of their data protection practices and will provide a number of advantages for sharing data.

## The enforcement of the GDPR

The Directive 95/46/EC is repealed with effect from 25 May 2018. During the two years of transition, any new processing should refer to the GDPR. Ongoing processing which have been authorised under the previous Directive remains valid. They should comply with the new GDPR in a two-year delay from May 2018. Member States will have a great role in revising their legislations on health research in order to comply with the GDPR and eventually specify its provisions according to national research policies. The new EDPB will also have a prominent role in specifying some aspects of the Regulation while the Commission retain certain competences for adopting implementation acts and technical documents at EU level.

## Conclusion

The GDPR preserves the equilibrium between the necessity of effectively protecting data subjects’ rights in a digitalised and globalised world while allowing the processing of personal data, including sensitive data, for scientific research. It reinforces cooperation duties and transparency between the actors of the processing, internally and with regard to the supervisory authorities, which should create a more integrated EU data protection system and diminish some useless administrative costs by decentralising elements of the data protection governance towards data controllers and processors. While the GDPR adopts new specific provisions to ensure adapted data protection in research, the field remains widely regulated at national level, in particular, regarding the application of research participants’ rights, which some could regret. However, the GDPR has the merit to set up clearer rules that will positively serve the research practices notably regarding consent, regarding the rules for reusing personal data for another purpose, assessing the risks of data processing in the context of DPIA, adopting accountable management system of processing operations and building or reinforcing internal data protection competencies with the DPO. In addition, for the first time, the GDPR refers to the respect of ethical standards as being part of the lawfulness of the processing in research, what must be saluted as an effort for sector-specific consistency. Finally, the GDPR opens new possibilities for going ahead in the structuring of data sharing in scientific research with measures encouraging self-regulation development. Optimistically, the news brought about by this new act should further structure the European Research Area and balance the necessary investments to be planned for reaching legal compliance.

## Notes

1 ‘Processing’ means any operation or a set of operations which is performed on personal data or on sets of personal data, whether or not by automated means, such as collection, recording, organisation, structuring, storage, adaptation or alteration, retrieval, consultation, use, disclosure by transmission, dissemination or otherwise making available, alignment or combination, restriction, erasure or destruction. See Article 4(2) of the GDPR.

2 ‘Personal data’ means any information relating to an identified or identifiable natural person (data subject); an identifiable natural person is one who can be identified directly or indirectly, in particular, by reference to an identifier such as a name, an identification number, location data, an online identifier or to one or more factors specific to the physical, physiological, genetic, mental, economic, cultural or social identity of that natural person; See Article 4(1) of the GDPR. Note that anonymous data that definitely not allow to identify the data subjects still be excluded from the scope of the GDPR, as it was under the previous Directive.

3 Article 8, Charter of Fundamental Rights of the European Union, see endnote n°3.

4 Article 16, Treaty on the Functioning of the European Union, see endnote n°4.

5 The ‘data subject’ is an identified or identifiable natural person to whom the personal data processed relate. Data subjects are the addressees of the rights set forth by the GDPR. See Article 4(1) of the GDPR.

6 See Recital 15 of the GDPR for more details on the concept of technological neutrality in the Regulation.

7 The ‘data controller’ is the natural or legal person, public authority, agency or other body which, alone or jointly with others, determines the purposes and means of the processing of personal data, where the purposes and means of such processing are determined by Union or Member State law, and the controller or the specific criteria for its nomination may be provided for by Union or Member State law. See Article 4(7) of the GDPR.

8 The ‘processor’ is a natural or legal person, public authority, agency or other body which processes personal data on behalf of the controller. See Article 4(8) of the GDPR.

9 ‘For the purposes of this Regulation, the processing of personal data for scientific research purposes should be interpreted in a broad manner including, for example, technological development and demonstration, fundamental research, applied research and privately funded research […].’ See Recital 159 of the GDPR. ‘Where personal data are processed for historical research purposes, this Regulation should also apply to that processing. This should also include historical research and research for genealogical purposes, bearing in mind that this Regulation should not apply to deceased persons.’ See Recital 160 of the GDPR.

10 Specific ethical issues in the realm of research are not tackled through the GDPR as it is not part of the competency of the EU to adopt laws on these matters; but it refers to ethical guidelines in the field as part of the normative environment of scientific research to consider with the rules fixed by the GDPR

11 See CHAPTER IX of the GDPR fixing ‘Provisions relating to specific processing situations’ and, in particular, Article 89 regarding the ‘Safeguards and derogations relating to processing for archiving purposes in the public interest, scientific or historical research purposes or statistical purposes.’

12 Defined as ‘personal data revealing racial or ethnic origin, political opinions, religious or philosophical beliefs or trade union membership and the processing of genetic data, biometric data for the purpose of uniquely identifying a natural person, data concerning health or data concerning a natural person’s sex life or sexual orientation’; specified definition, see Article 9 and Recital 51 of the GDPR.

13 This paper does not aim to be exhaustive. As an example, it does not address the news introduced by the regulation regarding the one-stop-shop mechanism, the powers and duties of the NDPA or ‘supervisory authorities’ and the remits of the European Data Protection Board (EDPB).

14 The implementing rules of the general principle of transparency are much more developed in Chapter III, Section 1 of the GDPR.

15 Principles of ‘lawfulness, fairness and transparency.’

16 Principle of ‘purpose limitation.’

17 Principle of ‘data minimisation.’

18 Principle of ‘data accuracy.’

19 Principle of ‘storage limitation.’

20 Events constituting a ‘personal data breach’ in the meaning of Article 4 (12), see infra.

21 ‘Research integrity’ may be defined as active adherence to the ethical principles and professional standards essential for the responsible practice of research. This notably includes a prohibition of fabrication, falsification and plagiarism and promotes honesty in research practices, confidential research data management as well as confidentiality and responsible sharing. Any breach of the research integrity rules is constitutive of research misconduct. See the Council of the EU Conclusions on Research Integrity, 2011, endnote n°5.

22 ‘Privacy enhancing technologies (PET) is a general term for a set of computer tools, applications and mechanisms which—when integrated in online services or applications, or when used in conjunction with such services or applications—allow online users to protect the privacy of their personally identifiable information provided to and handled by such services or applications.’ Wikipedia, accessed on 21 July 2016, endnote n°6. Authors also define PET as a ‘system of ICT measures protecting informational privacy by eliminating or minimising personal data thereby preventing unnecessary or unwanted processing of personal data, without the loss of the functionality of the information system.’ See Blarkom (2003), endnote n°7.

23 See Article 9(1) and Recital 51.

24 See Article 9(2) and Recitals 51–54.

25 Where it is necessary for the purposes of health or social care or treatment, of medical diagnosis, of preventive or occupational medicine, for the assessment of the working capacity of the employee or the management of health or social care systems and services, on the basis of Union or Member State law or pursuant to contract with a health professional. See Article 9(2)(h) of the GDPR.

26 Where the processing is necessary for reasons of public interest in the area of public health, such as protecting against serious cross-border threats to health or ensuring high standards of quality and safety of health care and of medicinal products or medical devices, on the basis of Union or Member State law which provides for suitable and specific measures to safeguard the rights and freedoms of the data subject, in particular professional secrecy. See Article 9(2)(i) of the GDPR.

27 See Article 9(2)(j) of the GDPR.

28 Article 4(15) of the GDPR.

29 Recital 35 of the GDPR.

30 Article 4(13) of the GDPR.

31 Recital 34 of the GDPR.

32 Article 4(14) of the GDPR.

33 Article 4(5) and Recitals 26 and 28 of the GDPR.

34 Recital 26 of the GDPR.

35 Article 4(11) of the GDPR.

36 Articles 32(a) and 2(a) of the GDPR, as amended in 2014 by the EU Parliament, ref. op.cit.

37 E.g. Erika Check Hayden (2013), see endnote 10. This article comes back on the previous demonstrations showing that reidentification of individuals through the use of their ‘anonymous-ised’ genome and cross-matching technics using publicly available information on the Internet and provides an overview of the challenges related to privacy protection in the context of genomic research.

38 Article 36 of the GDPR.

39 On the questions related to this notion, see supra, point on the Data Protection Officer.

40 Chapter VII, Section 2 of the GDPR on consistency.

41 This rule needs to be moderated as Article 36(5) states that ‘[…] Member State law may require controllers to consult with, and obtain prior authorisation from, the supervisory authority in relation to processing by a controller for the performance of a task carried out by the controller in the public interest, including processing in relation to social protection and public health,’ including where the result of a DPIA is not emphasising a ‘high risk.’

42 Article 35(11) of the GDPR.

43 Recital 77 of the GDPR.

44 ‘Personal data may be stored for longer periods insofar as the personal data will be processed solely for archiving purposes in the public interest, scientific or historical research purposes or statistical purposes in accordance with Article 89(1) subject to implementation of the appropriate technical and organisational measures required by this Regulation in order to safeguard the rights and freedoms of the data subject.’ Article 5(1)(b) of the GDPR.

45 Article 6(4) and Recital 50 of the GDPR.

46 Article 4(13) of the GDPR.

47 Recital 85 of the GDPR.

48 Article 33 and Recital 85 of the GDPR.

49 Article 34 and Recital 86 of the GDPR.

50 Article 33(3) of the GDPR.

51 E.g. where personal data are processed for archiving purposes in the public interest, see Article 89(3).

52 Article 6(1)(a) and regarding the processing of sensitive data Article 9(2)(a).

53 Recital 54 of the GDPR.

54 In particular, in the realm of research biobanking (collections of samples and data) where the long-run reusability of the biological resources is essential, knowing that broad consent practice is legally permitted and ethically admitted in some EU Member States such as in Sweden or Germany, but not in some others such as in France and Italy privileging a specific consent at the basis of the involvement of an individual in research, and specific procedures with the intervention of ethics committees or other kind of independent committees competent for assessing activities involving the reuses of the materials and approve them.

55 Recital 29 of the CTR.

56 Recital 33 of the GDPR.

57 Articles 40 and 41 and the following of the GDPR.

58 Article 47 of the GDPR.

59 Articles 42 and 43 of the GDPR.
